# Supplementary material for: Venous puncture wound hemostasis results in a vaulted thrombus structured by locally nucleated platelet aggregates
Source: Commun Biol. 2021 Sep 16;4:1090. doi: 10.1038/s42003-021-02615-y (PMC8445961; doi:10.1038/s42003-021-02615-y)
Supplement: Supplementary file 2 — Supplementary Information [file 42003_2021_2615_MOESM2_ESM.pdf]

# Supplementary Information

## Venous puncture wound hemostasis results in a vaulted thrombus structured by locally nucleated platelet aggregates

Sung Rhee[1],* Irina D. Pokrovskaya[2,*], Kelly Ball[1], Kenny Ling[3], Yajnesh Vedanaparti[3], Joshua Cohen[3], Denzel Cruz[3], Oliver Zhao[3], Maria A. Aronova[3], Guofeng Zhang[3], Jeffrey A Kamykowski[2], Richard D. Leapman[3], and Brian Storrie[2,+]

[1]Department of Pharmacology and Toxicology
University of Arkansas for Medical Sciences
401 West Markham Street
Little Rock, AR 72205
USA

[2]Department of Physiology and Cell Biology
University of Arkansas for Medical Sciences
401 West Markham Street
Little Rock, AR 72205
USA

[3]Laboratory of Cellular Imaging and Macromolecular Biophysics
National Institute of Biomedical Imaging and Bioengineering, National Institutes of Health
Bethesda, MD 20814
USA

*Both authors contributed equally

[+]Corresponding author, StorrieBrian@uams.edu)

**MOVIES**

Supplementary Movie 1: 1 min thrombus, fully rendered, initially shown as color transparent and then as solid color, SBF-SEM. Blue, vessel wall; green, tightly adherent platelets; orange, degranulated platelets; yellow, loosely adherent platelets.

Supplementary Movie 2: 5 min thrombus showing bleeding cessation via the extravascular cap and trapped RBCs on the intravascular side, SBF-SEM rendered, same thrombus as shown in Figure 2, Blue, vessel wall; red, RBCs; green, tightly adherent platelets; orange, degranulated platelets; yellow, loosely adherent platelets.

Supplementary Movie 3: 5 min thrombus, fully rendered, shown transparent and solid rendered, SBF-SEM, Segmented transparent colors give better visualization of the internal complexity of the thrombus, color scheme as before.

Supplementary Movie 4: 1 min thrombus, platelet accumulation through full puncture hole depth, Showing pedestals as binned EM and vessel wall as rendered, blue color, SBF-SEM. Images start on extravascular side and progress to intravascular side.

## SUPPLEMENTARY FIGURES

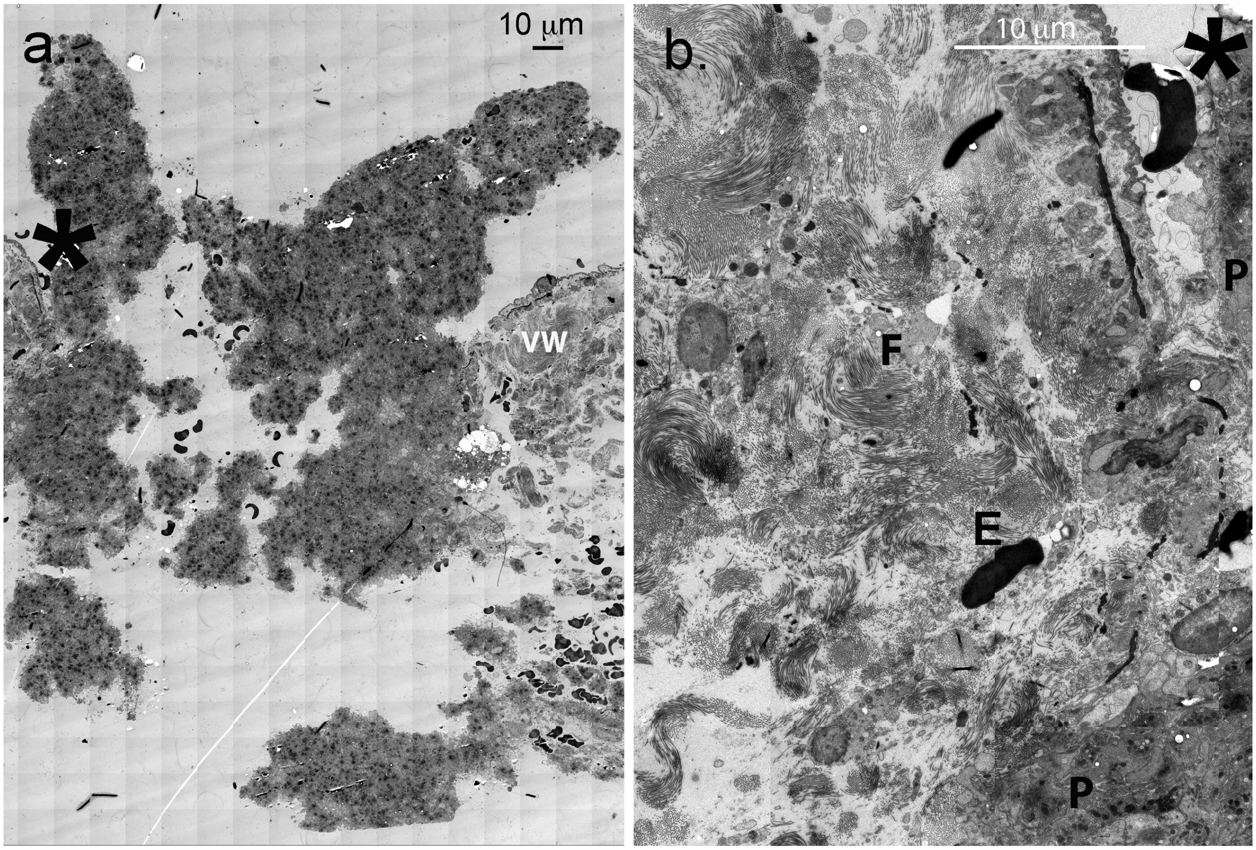


**Supplementary Figure 1.** 3 nm imaging reveals collagen fibrils about the exposed adventitia lining a jugular vein puncture hole. a. Low zoom image showing a WA-TEM image of a montaged, autofocused, cross-section of a 1-min thrombus. Asterisk in “a” indicates the area shown in “b” at a higher zoom. VW (vessel wall, a), lined by endothelial on its upper surface and an underlying adventitia that is rich in bundled fibers that show the morphological traits expected of collagen (see, b). b. zoomed image from the asterisk marked area in “a” showing fibers either end on (E) or en face (F). The bundling of the fibers and the variation in electron density along the fiber lengths are characteristic of collagen. P = platelets. Raw image size: ~90,000 pixels x 130,000 pixels.

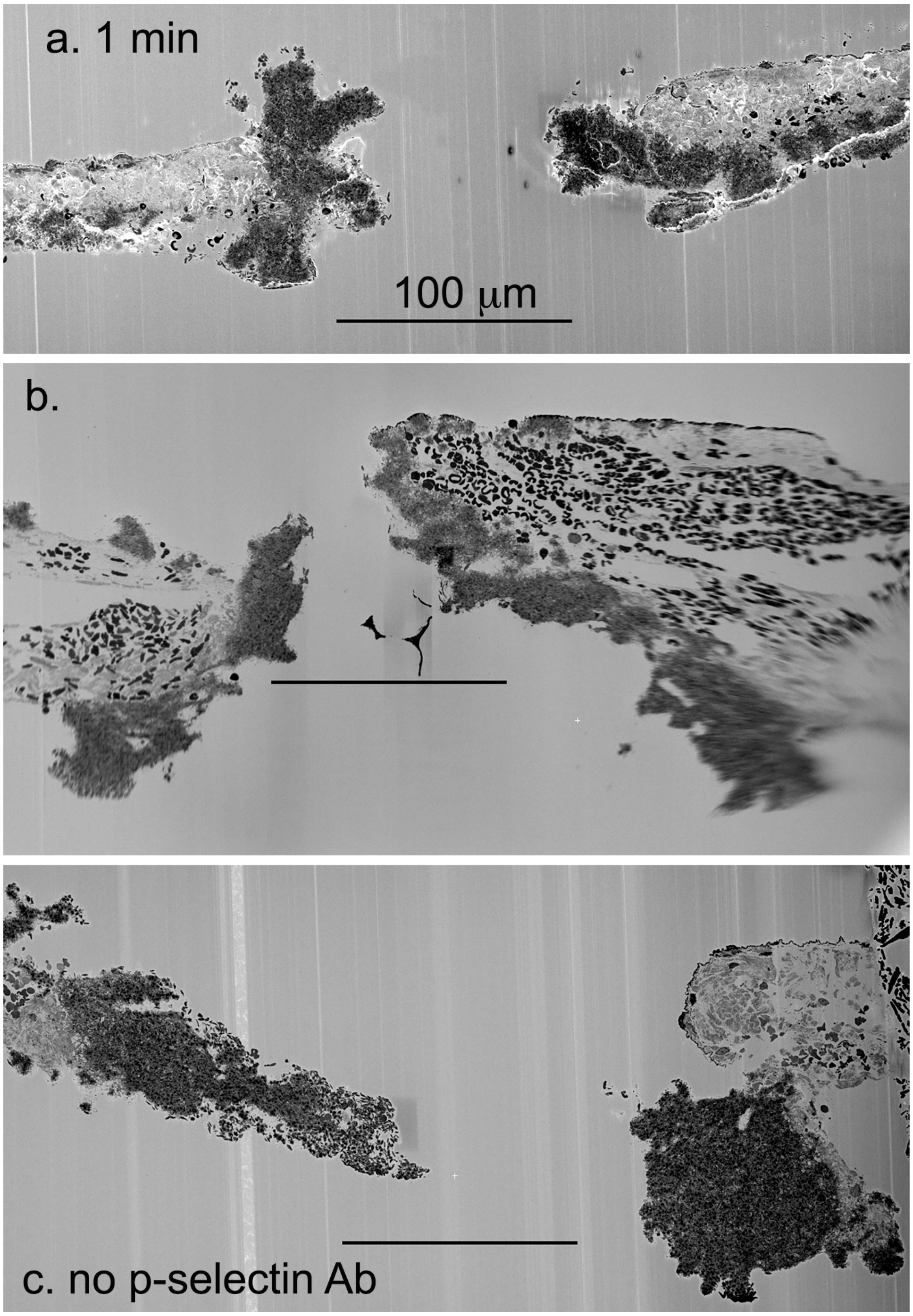


**Supplementary Figure 2.** Additional ultrastructural examples of 1 min jugular vein thrombi**.** a/b. Prepared after full 3 antibody infusion, i.e., anti-CD41, fibrin, p-selectin. Each image shows a single slice, mid-thrombus, SBF-SEM visualization, 20 nm raw XY pixel size, 20 μm spacing. See Figure 2 for a fourth example. All examples have similar structure. c. 2 antibody infusion during preparation, no p-selectin antibody, mid-thrombus SBF-SEM image slice. The intravascular lumen of the mouse jugular vein is to the top and extravascular side of the vessel wall is to the bottom. The vein is lined by a layer of endothelial cells and collagen-rich adventitia below in these images.

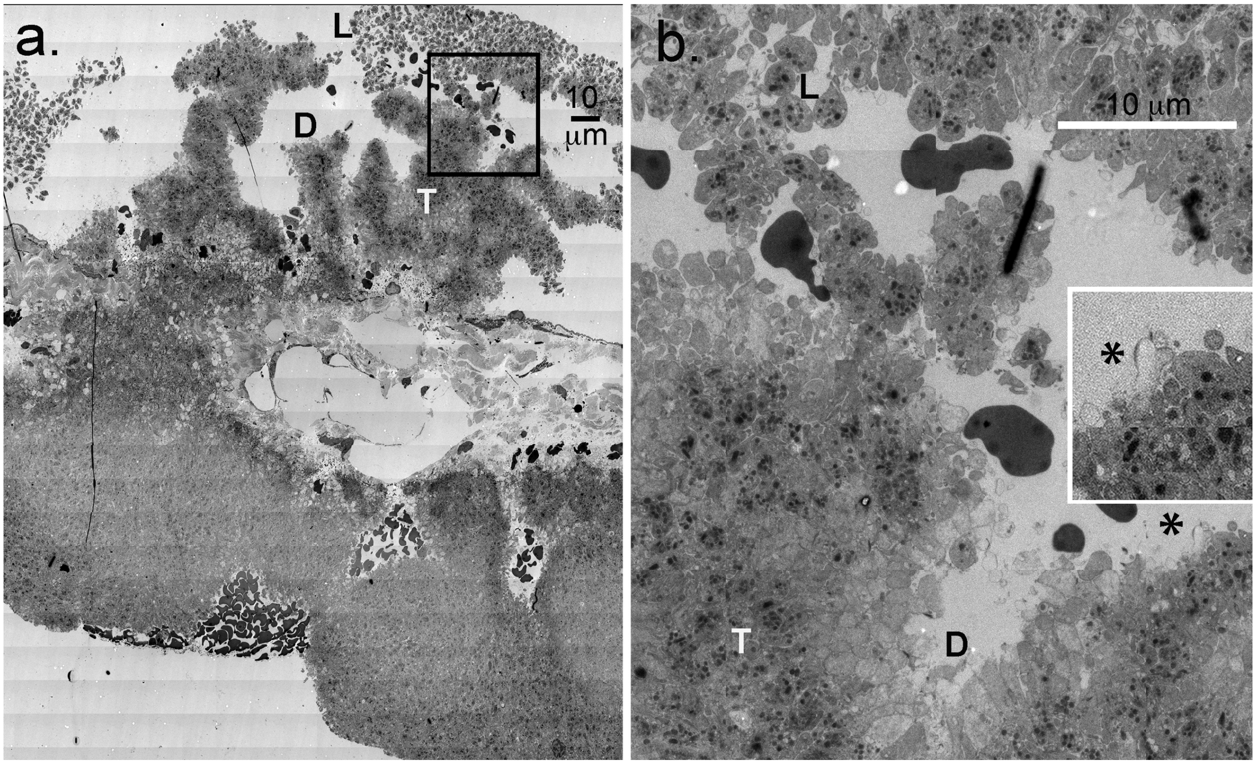


**Supplementary Figure 3.** 3 nm imaging provides more detailed, reference definition of the 3 platelet segmentation classes: loosely adherent (L), tightly adherent (T) and degranulated (D). a. Low zoom montaged WA-TEM cross section taken at a 3 nm XY pixel size. The raw image is approximately 90,000 x 130,000 pixels. b. Image of the boxed area in “a” shown at a higher zoom. Dark granules within L and T platelets can be readily seen within these segmentation classes. At a higher zoom, most of these granules can be shown to be $\alpha$-granules. The peripheries of the vaults areas are marked by degranulated platelets (D) which often show blebbing, a trait of procoagulant platelets. The inset to “b” shows in more detail blebbing of apparent procoagulant platelets. Asterisks mark area of inset and example blebbing. In segmentation analysis the degranulated platelets appear as light staining zones. As expected, at a 3 nm raw pixel size, the images can be zoomed further without pixilation becoming obvious.

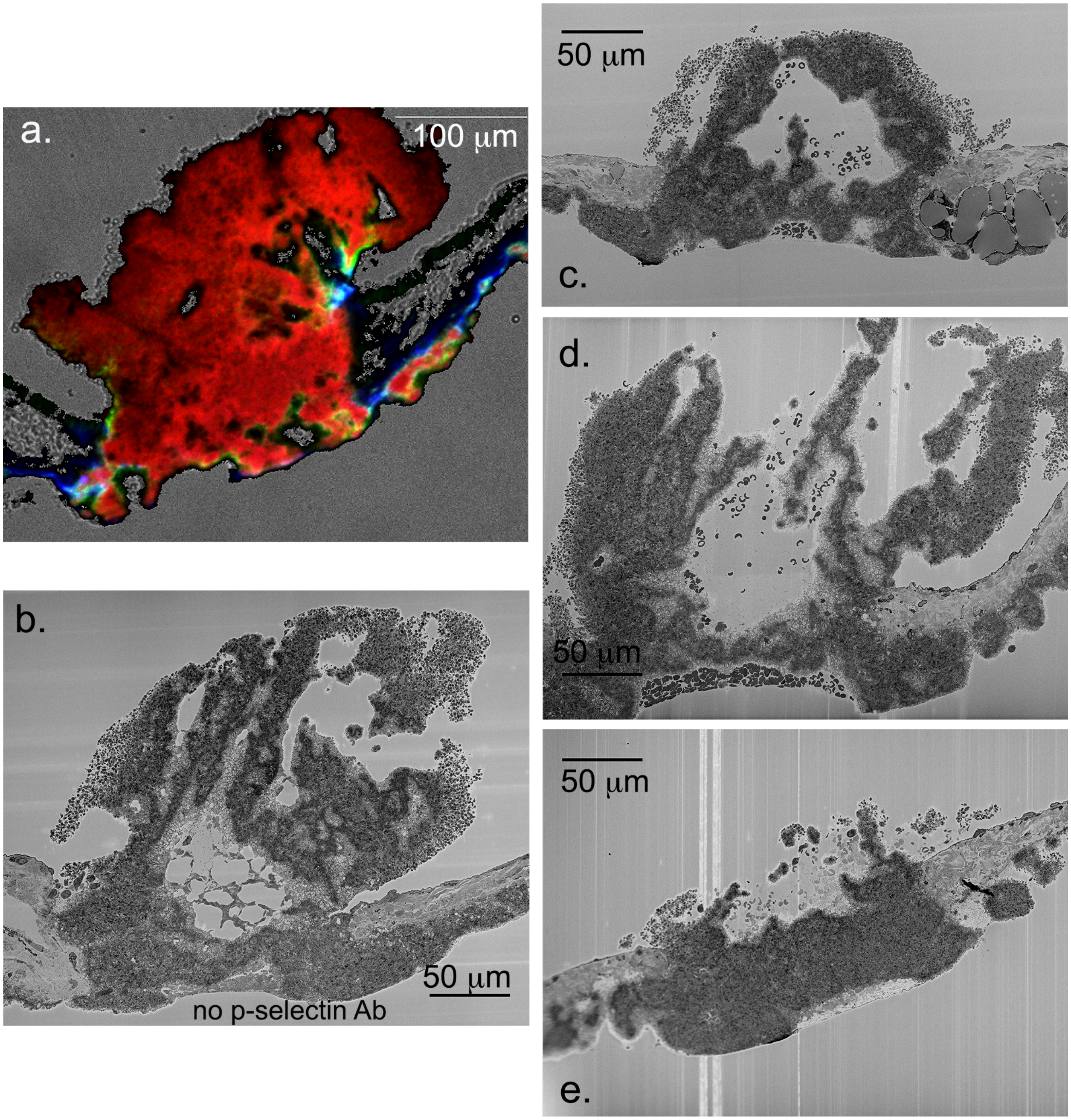


**Supplementary Figure 4.** Five min thrombus example controls. a. 2P image of fibrin, p-selectin, CD41 staining, ~~b.~~ mid-thrombus ultrastructure of a 2-antibody prepared sample (no p-selectin Ab infusion) and c-e. three additional, mid-thrombus, cross section examples of standard 3-antibody infused preparations. a. Frozen section image: Blue, fibrin staining, mostly extravascular; Green, p-selectin, variable patches towards the bottom, outer edges of thrombus; Red, CD41 staining; Light gray, vessel wall. b-e. SBF-SEM visualization, 20 nm raw XY pixel size, 20 μm spacing. See Figure 4 for a fifth example. All examples show a similar structural arrangement with an extravascular cap that does not entirely fill the puncture hole and a variable intravascular crown structured about vaults and columns. Zones of lightly stained platelets indicate areas of degranulation. These frequently line the outer surface of the columns. Note: The extent of RBC

entrapment within the vaulted regions of the thrombus depends on how closed the vault is on the intravascular side in these fixed, prefusion washed preparations.

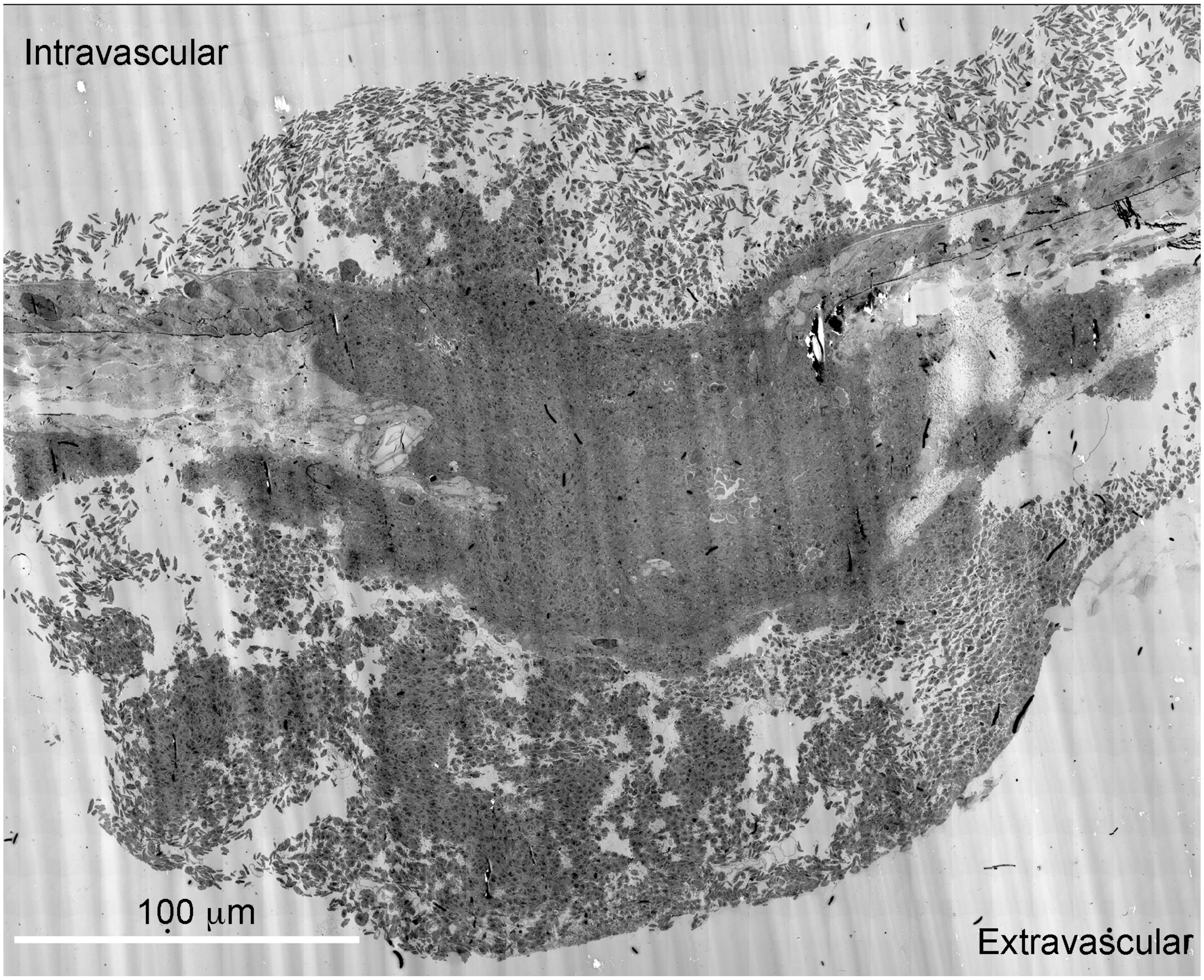


**Supplementary Figure 5.** A mouse femoral artery thrombus, 20-min post puncture, displayed predominantly extravascular platelet accumulation with apparent fragmentation of the platelet aggregates presumably due to rebleeding episodes. WA-TEM.
