# Supplementary material for: Venous puncture wound hemostasis results in a vaulted thrombus structured by locally nucleated platelet aggregates
Source: Commun Biol. 2021 Sep 16;4:1090. doi: 10.1038/s42003-021-02615-y (PMC8445961; doi:10.1038/s42003-021-02615-y)
Supplement: Supplementary file 3 — Description of Supplementary Files [file 42003_2021_2615_MOESM3_ESM.pdf]

Description of Additional Supplementary Files

File name: Supplementary Movie 1
Description: 1 min thrombus, fully rendered, initially shown as color transparent and then as solid color, SBF-SEM. Blue, vessel wall; green, tightly adherent platelets; orange, degranulated platelets; yellow, loosely adherent platelets.

File name: Supplementary Movie 2
Description: 5 min thrombus showing bleeding cessation via the extravascular cap and trapped RBCs on the intravascular side, SBF-SEM rendered, same thrombus as shown in Figure 2, Blue, vessel wall; red, RBCs; green, tightly adherent platelets; orange, degranulated platelets; yellow, loosely adherent platelets.

File name: Supplementary Movie 3
Description: 5 min thrombus, fully rendered, shown transparent and solid rendered, SBF-SEM, segmented transparent colors give better visualization of the internal complexity of the thrombus, color scheme as before.

File name: Supplementary Movie 4
Description: 1 min thrombus, platelet accumulation through full puncture hole depth, showing pedestals as binned EM and vessel wall as rendered, blue color, SBF-SEM. Images start on extravascular side and progress to intravascular side.

File name: Supplementary Data 1
Description: Source data for Figure 1b.

File name: Supplementary Data 2
Description: Source data for Figure 1c.

File name: Supplementary Data 3
Description: Source data for Figure 7b
